# Testosterone Replacement Therapy Prevents Alterations of Coronary Vascular Reactivity Caused by Hormone Deficiency Induced by Castration

**DOI:** 10.1371/journal.pone.0137111

**Published:** 2015-08-31

**Authors:** Wender Nascimento Rouver, Nathalie Tristão Banhos Delgado, Jussara Bezerra Menezes, Roger Lyrio Santos, Margareth Ribeiro Moyses

**Affiliations:** Department of Physiological Sciences, Federal University of Espírito Santo (Universidade Federal do Espírito Santo–UFES), Vitória, Brazil; Baylor College of Medicine, UNITED STATES

## Abstract

The present study aimed to determine the effects of chronic treatment with different doses of testosterone on endothelium–dependent coronary vascular reactivity in male rats. Adult male rats were divided into four experimental groups: control (SHAM), castrated (CAST), castrated and immediately treated subcutaneously with a physiological dose (0.5 mg/kg/day, PHYSIO group) or supraphysiological dose (2.5 mg/kg/day, SUPRA group) of testosterone for 15 days. Systolic blood pressure (SBP) was assessed at the end of treatment through tail plethysmography. After euthanasia, the heart was removed and coronary vascular reactivity was assessed using the Langendorff retrograde perfusion technique. A dose–response curve for bradykinin (BK) was constructed, followed by inhibition with 100 μM L-NAME, 2.8 μM indomethacin (INDO), L-NAME + INDO, or L-NAME + INDO + 0.75 μM clotrimazole (CLOT). We observed significant endothelium–dependent, BK–induced coronary vasodilation, which was abolished in the castrated group and restored in the PHYSIO and SUPRA groups. Furthermore, castration modulated the lipid and hormonal profiles and decreased body weight, and testosterone therapy restored all of these parameters. Our results revealed an increase in SBP in the SUPRA group. In addition, our data led us to conclude that physiological concentrations of testosterone may play a beneficial role in the cardiovascular system by maintaining an environment that is favourable for the activity of an endothelium–dependent vasodilator without increasing SBP.

## Introduction

Cardiovascular diseases (CVDs) constitute the most common cause of death worldwide, among which coronary artery disease assumes great importance [1]. Recent studies have shown that men are at higher risk of developing CVD compared with women of childbearing age, indicating a cardioprotective role of oestrogen [2,3], as demonstrated when cardioprotection ceases at menopause [2].

Because men are more susceptible to cardiovascular events, testosterone has been associated with deleterious effects [4,5]. However, other authors indicate that testosterone may have beneficial effects on the cardiovascular system (CVS) [6,7]. Therefore, studies on the activity of testosterone are inconclusive and often controversial.

Testosterone can exert rapid (“extra-nuclear”) vascular effects, including relaxation of the mesenteric bed [8] and mesenteric segment [9] in rats, relaxation of the aortic rings in rats [10] and pigs [11], and relaxation of the basilar artery in dogs [12].

It has also been shown that testosterone may act through both endothelium–dependent [13] and endothelium–independent mechanisms [11], where physiological concentrations would promote endothelium–dependent effects, whereas supraphysiological concentrations would lead to endothelium–independent effects [14,15].

The endothelium is a cellular monolayer lining the inside of blood vessels. Previous studies conducted in the 1980s reported that the endothelium plays a functional role in vascular reactivity [16]. This role is justified by the fact that the endothelium can respond to various physiological stimuli, even the simple friction caused by the passage of blood through the vessels (shear stress). Furthermore, the endothelium can respond to several substances circulating in the blood, including bradykinin [17]. Endothelial cells are important for the control of vascular tone through the production and release of endothelium–derived relaxing factors (EDRFs), such as nitric oxide (NO), prostacyclin (PGI_2_), and endothelium–derived hyperpolarizing factor (EDHF) [18–20].

While the direct action of testosterone on blood vessels has been well explored, the effects of chronic treatment on endothelium–dependent vascular function in the coronary circulation have not been fully elucidated. Claudio et al. [21] found that castration induced endothelial dysfunction in the coronary arteries of female rats. However, no reports are available regarding whether deprivation of testosterone through castration alters these parameters in male rats. Therefore, the aim of the present study was to evaluate the effects of castration and chronic testosterone treatment on endothelium–dependent vascular reactivity in Wistar male rats.

## Materials and Methods

### Experimental animals

In this study, 10-week-old adult Wistar rats (*Rattus norvegicus albinus*) were reared at the animal facility in the Centre of Health Sciences (Centro de Ciências da Saúde–CCS) at UFES. All procedures were approved by the Ethics Committee on Animal Use (CEUA) of UFES under protocol No. 049/2012. The animals were housed in cages (five animals per cage) and were provided food (Purina Labina) and water *ad libitum*. In addition, they were maintained under controlled conditions of temperature (22–24°C) and humidity (40–60%), with a 12/12 h light–dark cycle. The animals were randomly divided into four experimental groups: control (SHAM); castrated (CAST); castrated and treated with a physiological dose of testosterone (PHYSIO); and castrated and treated with a supraphysiological dose of testosterone (SUPRA).

### Castration

After anaesthesia with chloral hydrate (40 mg/kg, i.p.), the animals were placed in a supine position, fixed with surgical tape over a surgical bed. Before incision, disinfection of the testicular pouch was performed with iodized alcohol. Surgery was performed through an incision in the midline of the testicular pouch, and the testes were exposed by compression. After exposure of the testicles, the tunica vaginalis was opened, and the testes were ligated with absorbable sutures placed around the spermatic cord. Subsequently, the testes were removed, and the testicular pouch was sutured. The control group underwent sham surgery (SHAM) following the same procedures as the conventional surgery, except that the testicles were not removed. At the end of the procedure, the animals were given a dose of antibiotics (enrofloxacin at 2.5%, 0.1 mL, i.m.) to prevent infection.

### Hormone replacement

Testosterone replacement (bioidentical testosterone–IMAFAR) was performed for 15 days via subcutaneous injection of a dose of 0.5 mg/kg/day, mimicking a physiological concentration, as previously described [22], or 2.5 mg/kg/day, as a supraphysiological dose. The groups that did not receive testosterone replacement were administered the same volume of steroid vehicle (sunflower oil) [22]. The animals treated with testosterone received the first dose on the day of castration to avoid low hormonal levels post–surgery.

### Non-invasive assessment of blood pressure

After a period of adaptation, the animals were placed in a heated chamber within a container and restrained with a pneumatic cuff attached to the proximal region of the tail. A sphygmomanometer was inflated and deflated automatically, and SBP was recorded using a transducer coupled to a computer, as previously described [23]. The temperature was maintained between 29°C and 32°C for 40 minutes, during which the animals remained in the chamber (IITC INC/Life Science, 23924 Victory Blvd, Woodland Hills, CA 91367–1253 USA). An average of three measurements were obtained, with a maximum difference of 10 mmHg, and measurements associated with animal movements were discarded.

### Studies in isolated hearts

At the end of treatment, the animals were anesthetized with chloral hydrate (40 mg/kg, i.p.) and euthanized via decapitation for blood collection. The thoracic cavity was opened, and the heart was exposed, followed by removal of blood vessels. The heart was quickly transferred to the perfusion apparatus and isolated through cannulation of the aorta. Examination of the coronary vascular bed was conducted using the modified method of Langendorff (Hugo Sachs Electronics, March-Hugstetten, Germany). The isolated hearts were perfused with a nurturing solution containing 120 mM NaCl, 1.25 mM CaCl_2._2H_2_O, 5.4 mM KCl, 2.5 mM MgSO_4_.7H_2_O, 2.0 mM NaH_2_PO4.H_2_O, 27.0 mM NaHCO_3_, 1.2 mM Na_2_SO_4_, 0.03 mM EDTA, and 11.0 mM glucose, heated continuously at 37°C in a water bath and pressurized with a carbogenic mixture containing 95% O_2_ and 5% CO_2_ in a saturation chamber to maintain a stable pH of 7.4. Coronary flow was maintained constant at 10 mL/min using a roller pump (Hugo Sachs, Germany).

The baseline coronary perfusion pressure (CPP) was measured using a pressure transducer (AD Instruments MLT0380/A Reusable BP Transducer) connected in close proximity to the aortic perfusion cannula through which the coronary artery bed was perfused and connected to a digital data acquisition system (PowerLab System). Because the flow rate was maintained constant at 10 mL/min with a roller pump, the changes in CPP were directly related to changes in vascular resistance. A latex balloon at the end of a steel cannula was inserted into the left ventricle and connected to a pressure transducer (AD Instruments MLT0380/A Reusable BP Transducer) for measurement of the isovolumetric cardiac force. The balloon was pressurized with the aid of a glass syringe to maintain a preload of 10 mmHg.

After stabilization of the system for 40 min, baseline CPP was calculated, and a dose–response curve of bradykinin was generated (BK; Sigma, St. Louis, MO), which was administered in bolus form (0.1 mL) at increasing concentrations (0.1–1,000 ng) before and after perfusion with either 100 μM N^ω^-nitro-L-arginine methyl ester (L-NAME, a non-specific inhibitor of the enzyme nitric oxide synthase–NOS) or 2.8 μM indomethacin (INDO, a specific inhibitor of the enzyme cyclooxygenase–COX), or combined inhibition using either 100 μM L-NAME + 2.8 μM INDO or 100 μM L-NAME + 2.8 μM INDO + 0.75 μM clotrimazole (CLOT, an inhibitor of cytochrome P450–CYP).

At the end of the study, the heart was removed from the equipment, and the cardiac chambers were carefully separated and weighed on a precision scale (SHIMADZU AUY 220) after 24 hours of oven drying at 100°C to obtain the dry weight.

### Serological measurements

For determination of the lipid and hormonal profiles, 5 mL of blood was collected after decapitation. The blood was centrifuged (centrifuge Excelsa IV Model 280R) at 3,500 rpm for 15 minutes at 4°C, and the serum was collected and stored at –20°C. Assays for testosterone were performed based on electrochemiluminescence immunoassays using a Roche Diagnostics COBAS enzymatic kit. The assay sensitivity ranges were 0.025–15 ng/mL and limit of quantification were 0.120 ng/mL. The concentrations of total cholesterol (TC), triglycerides (TG), and high-density lipoprotein (HDL) cholesterol were measured using the enzymatic methods of the Colestat enzyme kit AA, TG Color GPO/PAP AA kit, and HDL cholesterol monophase AA Plus kit, respectively, with a Konelab model 600i spectrophotometer. The concentrations of low-density lipoprotein (LDL) cholesterol and very-low-density lipoprotein (VLDL) cholesterol were calculated using the Friedewald equation [24]: VLDL cholesterol = triglycerides/5, and LDL cholesterol = total cholesterol–(HDL + VLDL).

### Data analysis

Data analysis was performed using the statistical software GraphPad Prism 6. The data were expressed as the mean ± standard error of the mean (SEM). Comparisons between the groups were performed through one-way analysis of variance (one-way ANOVA), and the vasodilator response to bradykinin was evaluated through two-way analysis of variance (two-way ANOVA). In both cases, the post-hoc Tukey test was used, and a significance level of p<0.05 was established.

## Results

Table 1 shows that castration significantly decreased body weight compared with the SHAM group (from 358 ± 4 to 325 ± 7 g). However, treatment with testosterone prevented weight loss after castration in the PHYSIO (338 ± 7 g) and SUPRA groups (347 ± 6 g).

**Table 1:** Body weight and relationship of the dry weight of the heart chambers with body weight in the SHAM, CAST, SUPRA, and PHYSIO groups after 15 days of treatment with testosterone.

Parameter	N	SHAM	N	CASTRATED	N	PHYSIOLOGIC	N	SUPRA-PHYSIOLOGIC
**Body Weight (g)**	(30)	358 ± 4	(33)	325 ± 7*	(31)	338 ± 7	(32)	347 ± 6
**VD (mg/g)**	(16)	1,03 ± 0,03	(20)	1,06 ± 0,02	(24)	1,06 ± 0,03	(24)	1,04 ± 0,02
**VE (mg/g)**	(16)	3,99 ± 0,05	(20)	3,97 ± 0,05	(24)	3,97 ± 0,07	(24)	3,91 ± 0,06

The values are expressed as the mean ± SEM.

* p<0.05 compared with the SHAM group.

Castration did not alter the weight of the right ventricle (from 1.03 ± 0.03 to 1.06 ± 0.02 mg/g) or left ventricle (from 3.99 ± 0.05 to 3.97 ± 0.05 mg/g) compared with the SHAM group. Furthermore, the weight of the heart chambers was not altered by replacement with different doses of testosterone.

**Fig 1:**
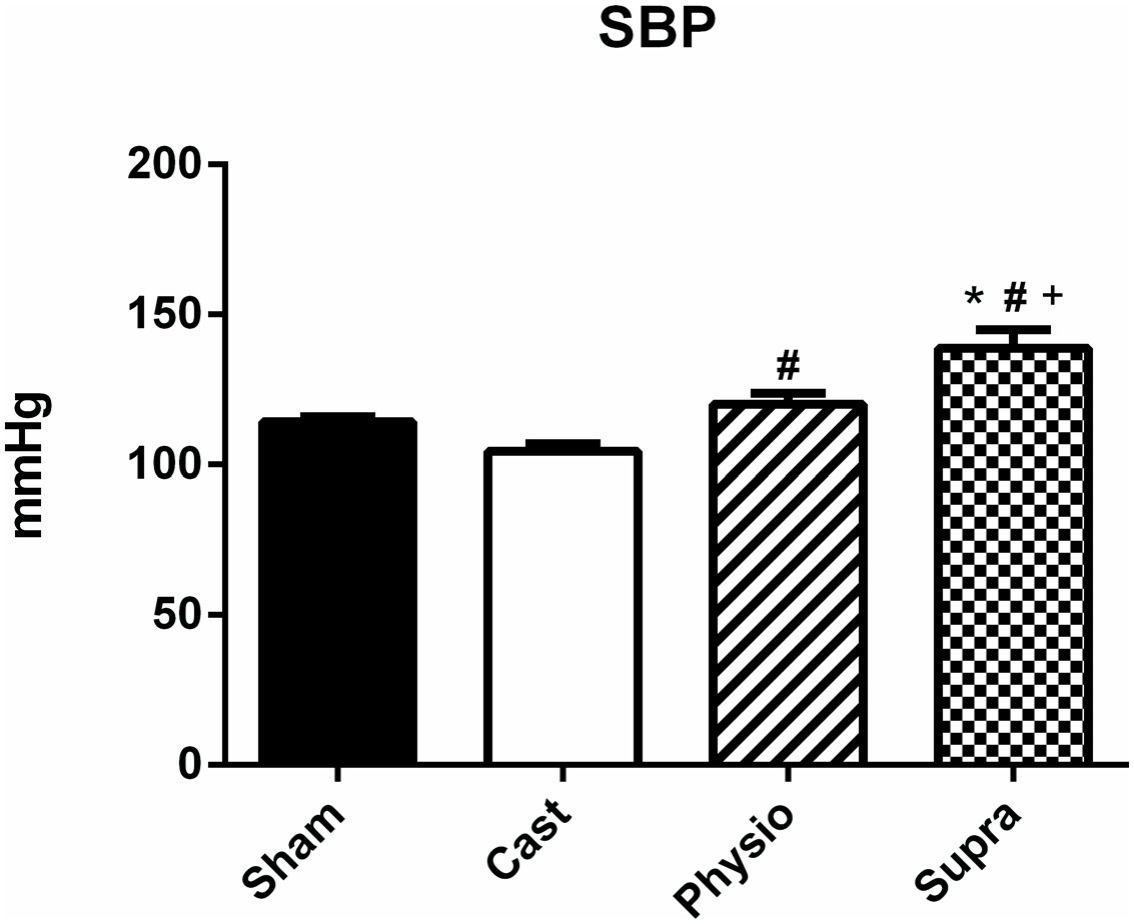
Systolic blood pressure of normotensive rats. SHAM (n = 15), CAST (n = 15), PHYSIO (n = 21), and SUPRA (n = 15) groups. The values are expressed as the mean ± SEM. * p<0.05 compared with the SHAM group, #p<0.05 compared with the CAST group, and +p <0.05 compared with the PHYSIO group.

Fig 1 shows the SBP values obtained in normotensive rats. Castration did not alter SBP compared with the control group (SHAM, 114 ± 2 mmHg, CAST, 104 ± 3 mmHg). However, treatment with a physiological dose of testosterone increased SBP (120 ± 4 mmHg) compared with the CAST group. As expected, SBP was significantly increased (139 ± 6 mm Hg) in the SUPRA group compared with the other groups.

**Fig 2:**
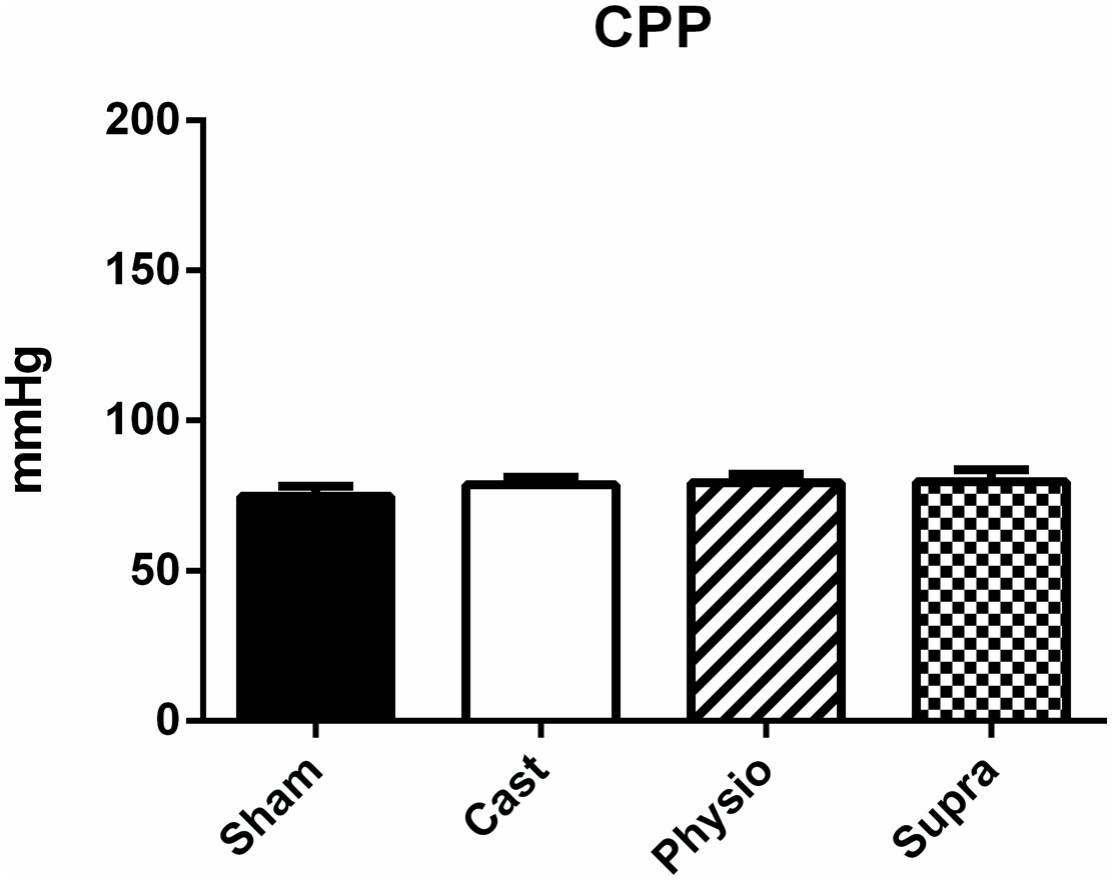
Baseline coronary perfusion pressure (CPP) of normotensive rats. SHAM (n = 20), CAST (n = 23), PHYSIO (n = 20), and SUPRA (n = 23) groups. The values are expressed as the mean ± SEM.

Unlike the changes observed for SBP, CPP was not altered in any of the study groups (SHAM, 75 ± 3 mmHg; CAST, 79 ± 3 mmHg; PHYSIO, 79 ± 3 mmHg, and SUPRA, 80 ± 4 mmHg), as shown in Fig 2. Therefore, the decrease in serum testosterone levels due to castration and the hormone replacement therapy applied in this study did not modulate the baseline coronary vascular tone.

Similarly, after adding the nurturing solution and inhibitors for the production of endothelium–derived relaxing factors, we observed a significant increase in CPP between the groups, although this difference was not significant, as shown in Table 2. Outside the aims of this study, the important role of endothelial mediators in maintaining coronary vascular tone can be observed in Table 2, especially with regard to the formation of NO and EDHF.

**Table 2:** Coronary perfusion pressure (CPP, mmHg) in normotensive rats after inhibition with 100 μM L-NAME, 28 μM indomethacin (INDO), L-NAME + INDO, or L-NAME + INDO + 0.75 μM clotrimazole.

Treatment	CONTROL		L-NAME		INDO		L-NAME + INDO		L-NAME + INDO + CLOT	
	N	mm/Hg	N	mm/Hg	N	mm/Hg	N	mm/Hg	N	mm/Hg
**SHAM**	(20)	75 ± 3	(09)	148 ± 9*	(09)	96 ± 11	(14)	144 ± 9*	(16)	158 ± 8*
**CASTRATED**	(26)	83 ± 4	(12)	149 ± 8*	(15)	110 ± 6*	(17)	151 ± 6*	(15)	167 ± 7*
**PHYSIOLOGIC**	(20)	79 ± 3	(08)	144 ± 5*	(15)	111 ± 9*	(11)	153 ± 8*	(11)	158 ± 15*
**SUPRA-PHYSIOLOGIC**	(23)	80 ± 4	(10)	137 ± 7*	(14)	105 ± 7*	(17)	152 ± 6*	(15)	153 ± 5*

The values are expressed as the mean ± SEM.

* p<0.05 compared with the respective controls.

**Fig 3:**
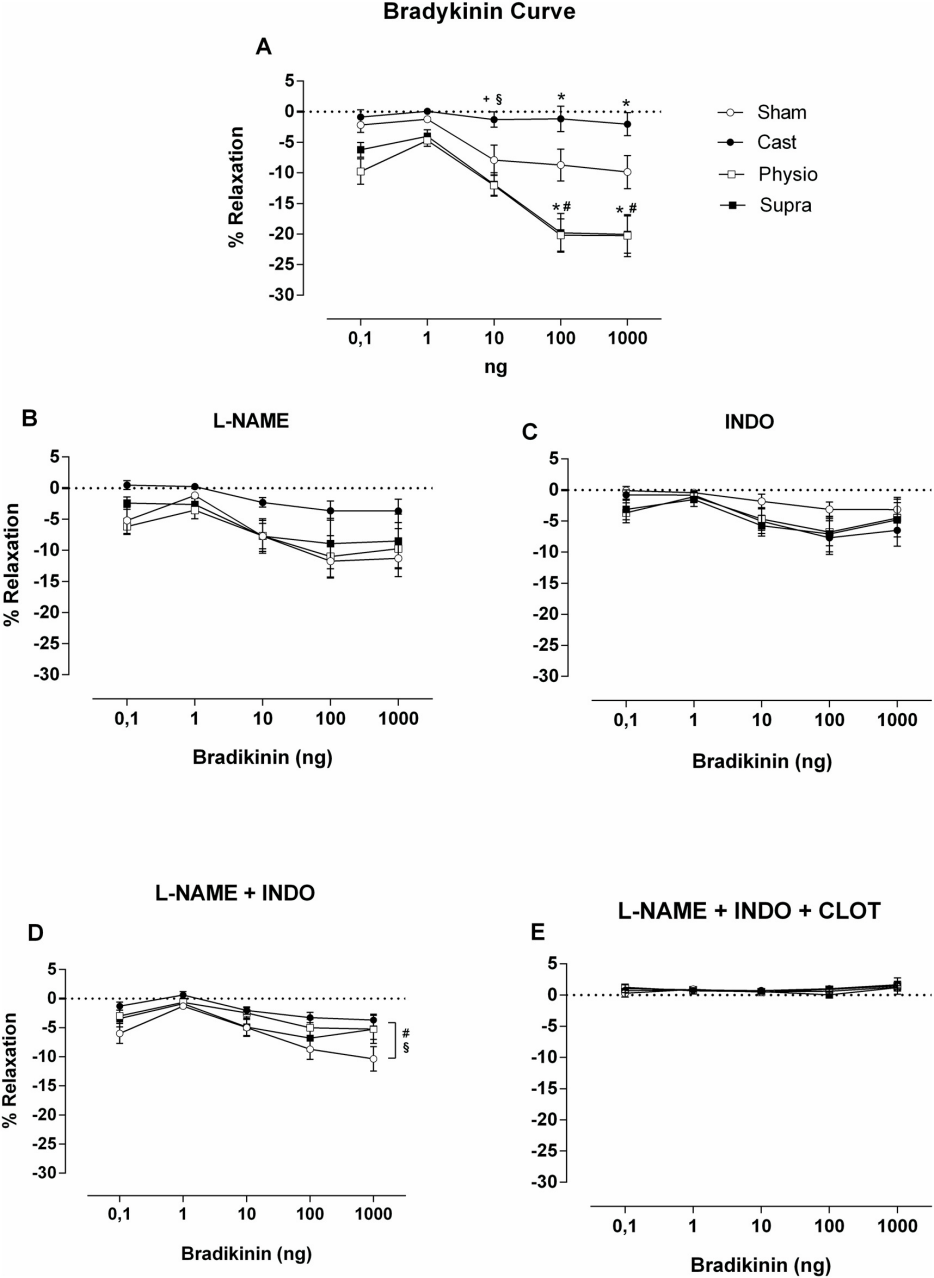
Vasodilator response to increasing concentrations of bradykinin (BK: 0.1–1,000 ng). Before (A) and after inhibition with L-NAME (B), indomethacin (INDO) (C); L-NAME + INDO (D), and L-NAME + INDO + clotrimazole (E). The values are expressed as the mean ± SEM. * p<0.05 compared with the SHAM group, #p<0.05 compared with the CAST group, +p<0.05 compared with the PHYSIO group, and §p <0.05 compared with the SUPRA group.

The main objective of this study was to evaluate the coronary vascular reactivity of normotensive rats after replacement treatment with different concentrations of testosterone. For this purpose, a relaxation curve was constructed using increasing concentrations of BK administered directly into the coronary vascular bed of rats (Fig 3).

As shown in Fig 3A, progressive relaxation occurred in the coronary vascular bed in response to BK in the SHAM group, and castration significantly impaired relaxation compared with the SHAM group. However, treatment with physiological and supraphysiological doses of testosterone prevented the impairment of relaxation caused by castration. Interestingly, both treatments not only prevented the impairment of relaxation observed in castrated rats but also enhanced BK-induced vasodilation compared with the SHAM group, suggesting that the presence of testosterone is necessary for bradykinin to exert its vasodilatory effects in this experimental model.

However, when NOS was inhibited with 100 μM L-NAME (Fig 3B), the vasodilator response decreased in the PHYSIO and SUPRA groups, so that the difference between these two groups was abolished compared with the SHAM group. The response of the SHAM group was not altered by L-NAME.

A similar result was observed when COX was inhibited with 2.8 μM INDO (Fig 3C). Inhibition of COX decreased BK-mediated relaxation, particularly in the PHYSIO and SUPRA groups, suggesting an important role of COX in this study model.

Combined inhibition with L-NAME + INDO (Fig 3D) further decreased relaxation in the PHYSIO and SUPRA groups to a level similar to that observed in the castrated group. Relaxation was significantly greater in the SHAM group than in the CAST group. Furthermore, the endothelium–dependent vasodilator response to BK treatment was completely inhibited in all of the evaluated groups when combined inhibition of NOS, COX, and CYP was performed (Fig 3E), indicating that the vasodilation detected in this study was most likely endothelium dependent.

**Fig 4:**
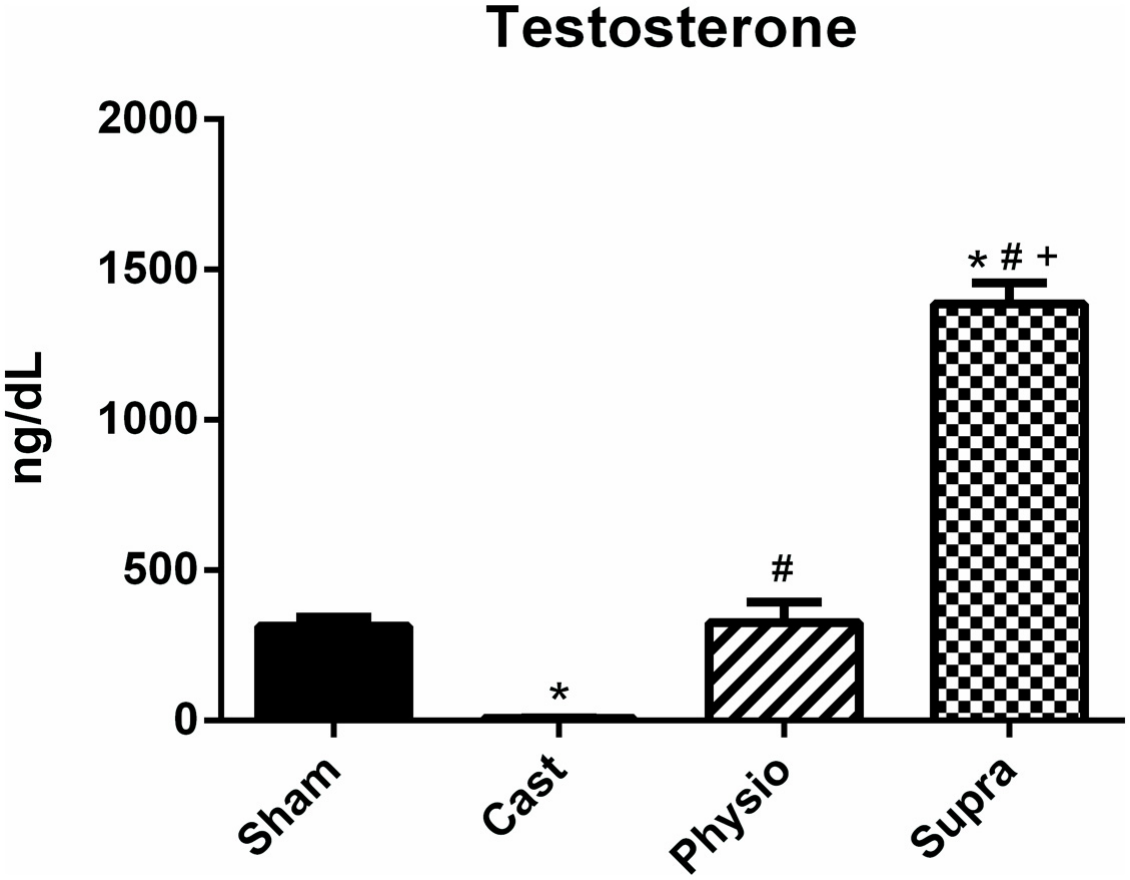
Hormone level. Testosterone in the SHAM (n = 15), CAST (n = 15); PHYSIO (n = 17), and SUPRA (n = 16) groups. Data are expressed as the mean ± SEM. * p<0.05 compared with the SHAM group, #p<0.05 compared with the CAST group, and +p<0.05 compared with the PHYSIO group.

By analysing the hormonal profile (Fig 4), we confirmed that castration was effective in significantly decreasing testosterone levels in the castrated group (SHAM, 314 ± 31 ng/dL; CAST, 4 ± 1 ng/dL). In addition, treatment with a physiological dose of testosterone (325 ± 69 ng/dL) was effective in maintaining a testosterone level similar to that observed in the SHAM group. On the other hand, the SUPRA group exhibited a marked increase in testosterone levels (1,385 ± 71.1 ng/dL).

**Fig 5:**
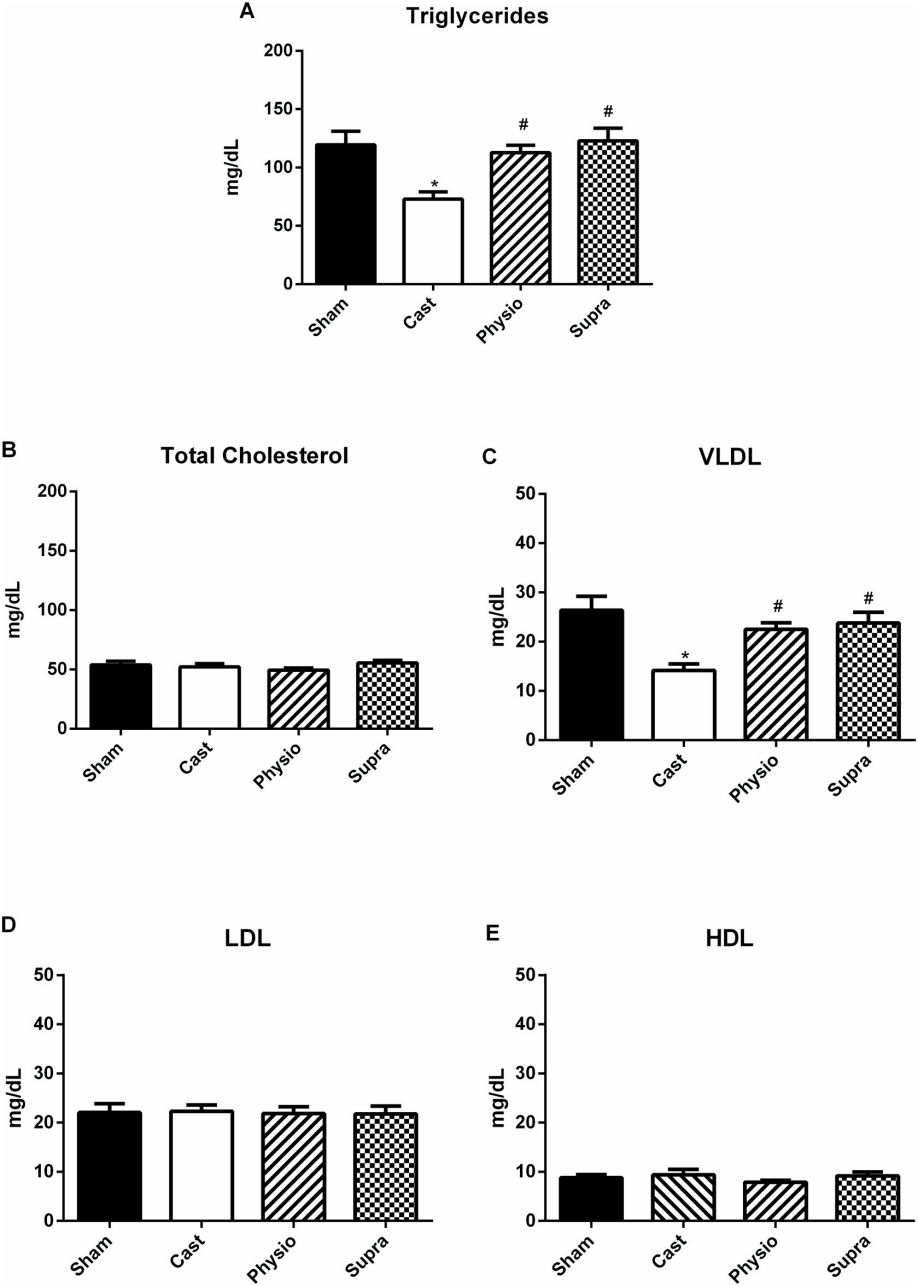
Lipid profile of normotensive rats. A) Triglycerides, B) total cholesterol, C) very-low-density lipoproteins (VLDL), D) low-density lipoprotein (LDL) cholesterol, and E) high-density lipoprotein (HDL) cholesterol in the SHAM (n = 15), CAST (n = 17), PHYSIO (n = 17), and SUPRA (n = 16) groups. Data are expressed as the mean ± SEM. *p<0.05 compared with the SHAM group, #p<0.05 compared with the CAST group.

Fig 5 shows that triglyceride levels were significantly decreased in the CAST group compared with the SHAM group (from 120 ± 12 to 73 ± 6 mg/dL). Hormone replacement at a physiological dose (113 ± 6 mg/dL) or supraphysiological dose (123 ± 11 mg/dL) restored triglycerides to levels similar to those found in the SHAM group (Fig 5A). Similarly, VLDL levels were significantly decreased in the CAST group (14 ± 1 mg/dL) compared with the SHAM group (26 ± 3 mg/dL) (Fig 5C) and a physiological dose (22 ± 1 mg/dL) or supraphysiological dose (24 ± 2 mg / dL) of testosterone restored VLDL levels after castration. Total cholesterol, LDL cholesterol, and HDL cholesterol remained unchanged in both groups (Fig 5B, 5D and 5E).

## Discussion

The main finding of this study was the observed efficacy of treatment with physiological and supraphysiological doses of testosterone in preventing the damage caused by hormone deficiency in the endothelium–dependent coronary vasodilation induced by BK. Furthermore, castration was found to alter lipid and hormonal profiles and decrease body weight. However, replacement with testosterone restored all of the above parameters to levels similar to those found in the SHAM group. We also observed an increase in SBP in the group treated with a supraphysiological dose of testosterone.

Our results indicate that body weight had decreased 15 days after castration compared with the SHAM group and that replacement therapy prevented this decrease. These data corroborate the results obtained by Bourghardt et al. [25], who also recorded a decrease in body weight after eight weeks of castration. However, previous studies in our laboratory [26] have indicated that testosterone replacement, even at high doses, fails to alter body weight compared with control animals.

One possible explanation for the weight loss recorded in the CAST group may be related to decreased serum levels of triglycerides. Our results indicate that the concentrations of triglycerides and VLDL (responsible for the transport of triglycerides in the blood) were decreased after 15 days of castration. Moreover, testosterone decreases the activity of the enzyme lipoprotein lipase (LPL) [27], and modulation of LPL may contribute to changes in adipose tissue, especially in men [28].

Considering that LPL is associated not only with the hydrolysis of triglyceride molecules found in plasma lipoproteins, chylomicrons, and VLDL [29], but also with adipogenesis, obesity, the energy balance, metabolic disorders, and abnormal regulation of body weight [30], the decreased inhibition of LPL exerted by testosterone after two weeks of castration may explain the weight loss observed in these animals. Nevertheless, because our data are inconclusive, further studies are required to elucidate the mechanisms associated with the activity of testosterone and the body composition, particularly with regard to the modulation of body weight.

Our results also confirm previous results with respect to SBP, where castration and replacement therapy with physiological doses of testosterone were not found to affect SBP. However, SBP levels increased significantly in the SUPRA group. In this respect, other studies have shown that high doses of testosterone can increase blood pressure [31], whereas castration does not alter SBP in normotensive rats [32].

It is well established that testosterone can stimulate the renin–angiotensin–aldosterone system (RAAS), which, through the formation of angiotensin II, can raise blood pressure [33]. Reckelhoff et al. [34] described the important role of RAAS in increasing blood pressure in spontaneously hypertensive rats and reported that RAAS was mainly influenced by testosterone. However, this influence was attenuated when an androgen receptor (AR) antagonist was administered [35], indicating that testosterone plays an important role in blood pressure regulation.

It has been shown that testosterone can modulate the metabolism, storage, release, and reuptake of norepinephrine in sympathetic neurons [36], which could potentially raise blood pressure. Furthermore, recent studies have provided evidence that administration of testosterone at supraphysiological doses can directly promote vasoconstriction in vascular smooth muscles [14,15]. Taken together, these findings support the hypothesis that testosterone modulates blood pressure, especially at high concentrations, as demonstrated herein.

Although SBP was modulated by testosterone, such modulation was not observed for CPP. Our results allow us to suggest that coronary vascular tone in normotensive rats may not be modulated by testosterone, in contrast to findings obtained for female hormones (oestrogen), as previous studies from our laboratory have demonstrated that castration decreases CPP in normotensive female rats [22].

Although we did not observed changes in CPP in the experimental groups, the primary objective of this study was to evaluate endothelium–dependent vascular coronary reactivity mediated by BK. Our results clearly indicate that testosterone treatment was effective in preventing the impairment of vasodilation caused by castration compared with the SHAM group. Moreover, both the physiological and supraphysiological treatments not only prevented this decrease but also enhanced vascular coronary relaxation.

The results of this study demonstrate for the first time that testosterone can modulate vascular reactivity in the endothelium–dependent coronary vascular bed in male rats. It is known that testosterone can directly modulate the CVS by relaxing vascular smooth muscle [8–12]. Notably, our results demonstrate the role of endogenous testosterone in maintaining an environment that is favourable for the action of a vasodilator agonist that acts on the vascular endothelium.

Other authors have shown that castration can decrease the expression of voltage–gated potassium channels [32], which in turn decreases potassium efflux and impairs vascular relaxation, as demonstrated by Ramires-Roses et al. [12]. However, the mechanism by which testosterone promotes the action of an endothelium–mediated vasodilator has not been fully elucidated.

In fact, our data suggest that castration somehow altered the vascular environment, possibly promoting endothelial dysfunction to the point that the animals exhibited an impaired response to BK. Previous studies have shown that sex hormones are involved in maintaining a healthy vascular environment. Accordingly, Claudio et al. [21] observed that the coronary reactivity of female rats was impaired by ovariectomy, and oestrogen replacement therapy restored these effects. Furthermore, high doses of testosterone can promote endothelial dysfunction by decreasing the expression of eNOS [37], which is the main source of endothelial NO [38]. Considering these results, further studies are required to elucidate the role of testosterone in the modulation of blood vessels.

Because the animals treated with testosterone experienced a degree of relaxation superior to that in the control animals, our objective was to evaluate the role of the endothelial mediators through which this relaxation occurred. For this purpose, the first step was to inhibit NOS with L-NAME (a non-specific NOS inhibitor). The result of this experiment revealed that incubation with L-NAME decreased BK–induced relaxation in the FISIO and SUPRA groups to levels similar to those found in the SHAM group, indicating the important role of NO in the dilation of the coronary arteries. In fact, testosterone can exert direct action on the coronary arteries by promoting vasodilatation, and this response often involves the formation of endothelial NO (via eNOS) and/or extra-endothelial sources of NO (via nNOS) [11]. These findings lead us to believe that, in addition to the direct action of testosterone on the vascular system, testosterone can maintain a favourable environment for the action of a vasodilator agonist, as observed herein.

BK is a potent endothelium–dependent vasodilator peptide that acts via stimulation of endothelial B_2_ receptors, promoting the release of NO, PGI_2_, and EDHF [39–41]. Accordingly, our results corroborate previous findings because, in addition to the importance of NO in the coronary circulation, as demonstrated herein, the animals with circulating testosterone experienced an impairment of the relaxation induced by BK via inhibition of cyclooxygenase (with indomethacin), which is the enzyme responsible for the formation of vasodilator prostanoids. The same effect could be observed when combined inhibition was performed using L-NAME and INDO, especially in the PHYSIO and SUPRA groups, confirming the role of NO and PGI_2_ in relaxation. Previous studies from our laboratory have demonstrated the importance of vasodilator prostanoids in normotensive male rats compared with other vasodilator agonists [42].

Furthermore, as expected, the vasodilator response to BK in the coronary vascular bed of normotensive rats was completely abolished in all groups when the three endothelial mediators were inhibited. In this case, clotrimazole (an inhibitor of cytochrome P450) was added along with L-NAME and INDO. These data provide evidence of the important role of EDHF in BK-induced relaxation. The involvement of CYP metabolites, i.e., epoxyeicosatrienoic acids, as candidate EDHFs in coronary arteries has been demonstrated in previous studies from our laboratory, in both normotensive [42] and hypertensive animals [43].

In summary, our data suggest that testosterone treatment can prevent damage due to hypogonadism in the studied model. Therefore, we suggest that the damage on the coronary vascular reactivity induced by extreme deficiency of male sex hormone, i.e. after castration, can be prevented by replacement of testosterone
